# Supplementary material for: ChimpanSEE, ChimpanDO: Grooming and play contagion in chimpanzees
Source: PLoS One. 2024 Nov 20;19(11):e0312467. doi: 10.1371/journal.pone.0312467 (PMC11578526; doi:10.1371/journal.pone.0312467)
Supplement: Attachment — Submitted filename: Response to Reviewers.docx [file pone.0312467.s003.docx]

REVIEWER 1

“ChimpanSEE, ChimpanDO:First evidence of grooming and play contagion in chimpanzees” is an interesting research article investigating groom and play contagion in 41 captive chimpanzees housed in a sanctuary in Zambia. The Authors used a well-suited PO-MC method to study whether chimpanzees show i) grooming/play contagion in 5 minutes after the observed grooming/play bout, and ii) whether this was dependent on their social closeness to the individual exhibiting grooming or play, or their age, sex or rank in the dominance hierarchy. The Authors compared the matched controls (MC), i.e. observations matched in time and social conditions, with the observations following grooming or playing bouts (PO). The Authors ask a timely question, use appropriate observational methods to study this question, analyse the data with modern statistical methods and discuss the findings appropriately, placing the study in the context of research on behavioural contagion and empathy. Overall, it was a pleasure to read this nice work. However, I also have some questions relating to the study design, analyses, and the interpretations of the found effects. These points are listed below in their appearing order, separated for major points, minor points and copy-edits, and I hope they will be helpful for the Authors in revising their work.

Major points

The Authors nicely explain how the grooming or play observations were defined (“We determined whether observation happened by considering the head orientation of the observer, and their distance to the behaviour. When the trigger was within 5-metres, and happened in the 180 in front of their face and in direct visual contact, we considered the behaviour as observed. PO focals therefore started either when a grooming or play interaction started within the subject’s observational area, or when a subject moved so that they observed the behaviour.”). Thus, I assume that the focal observers were only those individuals that did not participate in the grooming or play bout themselves. However, this has not been explicitly stated in the text, so it could be that Authors also considered event participants in the analyses. If the Authors considered both the individuals that were in close proximity and observed the event (yet not included in the event themselves) and also the participants in the grooming or play event (those that did not initiate the event, but participated in it) - this is then problematic. These events are reciprocal in nature, and the receivers of grooming or play usually initiate grooming/play afterwards with the same grooming/play partner afterwards, and this would not be considered contagion. Please elaborate and if I am (hopefully) wrong, include the information that the observers did not participate in the event itself.

*Thank you very much for pointing out the omission in the explanation here. We did indeed not include those involved in the play as observer focals, and have added clarification of this in lines 173-174:
‘Individuals who participated in the first play/grooming bout, even if they did not initiate it, were not included as observers, as they were already involved in the behaviour’.*

It is a bit confusing why the Authors only compared “attracted” and “dispersed” pairs, when in fact the “neutral” pairs made most of the pairs? How do Authors explain and deal with this big number of “neutral” pairs (e.g. L307: “N = 29 were attracted, N = 1 was dispersed, and N = 90 were neutral”, L320: N = 33 were attracted, N = 1 was dispersed, and N=62 were neutral)? This should be discussed in the text.

*Thank you very much for raising this point. We chose the analysis of attracted vs dispersed pairs based on previous literature, however we realise that in our dataset there is a large number of neutral pairs, and so excluding the neutral pairs may be inappropriate in this case. We have instead now carried out a Friedman test, with post-hoc pairwise comparisons using Wilcoxon tests with Bonferroni corrections, to compare attracted vs dispersed vs neutral pairs, so as to include all the data (lines 257-258; 317-325; 335-343).*

The review on behavioural contagion in animals (L64-74) is quite short and leaves the reader wondering about other literature. There are many more instances of contagious behaviour, including yawning, scratching, scent-marking, etc. in animals, but these are not mentioned here.

*Thank you for this point. We feel that, given the very large literature on behavioural contagion in animals, it is beyond the scope of this paper to review in full. We realise the importance of this research, and have added a sentence summarizing additional avenues and directing the reader to a review piece:
‘Many other behaviours have also been shown to be contagious- including vigilance in Japanese macaques (7), scent-marking in marmosets (27), and self-scratching and yawning in many different species (for review, 3).*

Minor Points.

L20. “contagion effect” is a bit unclear, please try to rephrase.
*Thank you, we have rephrased this to ‘behavioural contagion’.*

L24. “and that it is crucial to consider behavioural contexts when determining predictors of contagious behaviour” -> which behavioural contexts and how does this statement follow from your results? Please either elaborate or delete.
*Thank you for pointing out the ambiguity; we have reworded this phrase to ‘and that the predictors of contagious behaviour are highly specific to the behaviour and species in question’.*

L51-52. “activity reminiscent of serious functional contexts” -> do you mean that these behaviours are potentially functionally relevant for these animals?

*Thank you- we included this sentence to make a point of the breadth of behaviours that are considered playful, and have reworded it to make this point clearer:
‘Play includes a wide variety of activity; both behaviours that are reminiscent of serious functional contexts (e.g., fighting and mating behaviour), and of actions that have no immediate function or benefit’.*

L58-62. The play is mentioned in a non-positive context here, which leaves the reader wondering why was play then chosen as one of two ‘positive’ behaviours in this study. I would suggest moving this part to earlier in text and ending the paragraph on a more positive note about play.
*Thank you for the suggestion, we have re-ordered this section so that the paragraph ends with the description of the affiliative functions of play.*

First sentence of this paragraph (L76-77) should be omitted as this was already mentioned in the previous paragraph.
*Thank you- we have deleted this sentence.*

L79-83 are a bit broad - consider shortening and targeting more to this study.

*Thank you for this suggestion. We would like to keep in the point that research like this can elucidate aspects of evolution, as we feel it is important. However, we have reworded and slightly shortened the sentence for simplicity:
‘Further research on social contagion can enable cross-species comparisons with humans and other primates, which are key to detecting socio-cognitive and behavioural differences, and marking shifts in the evolution of hominin sociality.’*

L102, L119. To my reading, you did not measure the strength of the contagion effect, so consider rephrasing, or explain how the strength was measured.
*Thank you, we have changed this to specify ‘presence and latency’.*

L119-126. Would suggest rewriting this paragraph for clarity.
*Thank you for highlighting that this is not clear. We have reworded several sentences in this paragraph:
‘Research on facial mimicry and yawn contagion indicates that contagion mechanisms are present from infancy in chimpanzees, although some studies have found stronger effects in older individuals (for review: 3). Contagion of affiliative behaviours may show distinct patterns, as initiating a behaviour is a multicomponent process involving social cognition, and its expression is under voluntary control.’*

Data analysis. I would suggest to delete subtitles phrased as questions.
*Thank you for the suggestion. As this is a stylistic recommendation, we would prefer to keep in these subtitles, as we feel they add structure and provide continuity between the data analysis methods and results.*

L196-203. Description of how the metrics and the dyadic sociality index were calculated is unclear, please elaborate. Furthermore, was this data later used and how?
*Thank you, we have reworded this section to add more detail, and have added clarifications for how and when this data was used. The section now reads:
‘We calculated the proportion of time that each dyad spent engaging in each interactive behaviour (playing, grooming, contact sitting, proximity sitting), by dividing the number of scan-points they engaged in the behaviour by the total number of scan-points where at least one chimpanzee in the dyad was in view. The metrics of each behaviour correlated, and so we integrated them into an overall DSI. We divided each metric by the average of that metric across all dyads, and averaged the 4 scores for each individual (24,51). The DSI scores were entered into the GLMMs.
We also used this social data to compute scores for each individual’s overall grooming tendency and play tendency, by dividing the number of scan-points they respectively groomed and played in, by the total number of scans the individual was present for. These grooming/play tendency scores were entered into the GLMMs.’*

L220-222. “we excluded all observations from that point on, as it would not be possible to determine whether the focal’s subsequent behaviour was driven by their experience observing or engaging in the behaviour.” I do not follow this sentence, could you please elaborate further, and how this impacted the duration of total time that the individual was observed for, did you control for this discrepancy in the analyses?
*Thank you for pointing this out. We did not include these post-observation follows at all in the GLMM analysis. We have reworded this sentence to clarify this, and the rationale behind this. The sentence now reads:
‘If another individual initiated a grooming or play bout with the focal before the focal initiated a behaviour, this post-observation follow was excluded from the GLMM analyses, as it would not be possible to determine whether the focal’s subsequent behaviour was driven by their experience observing or engaging in the behaviour.’*

L225-227. Here it says that latencies were expressed as proportions of the 5-minute focal follow, but in the analyses we see the latencies expressed in seconds. Can you please explain this, and correct if/where necessary?
*Thank you for raising this. Data were analysed in the GLMMs as proportions of the 5-minute follow, but we included descriptive statistics of the raw data in seconds, to aid interpretation of the overall pattern of data. We have added clarification that this transformation of the latency data was ‘for the purpose of analysis in the GLMMs.’*

L253-255. These lines should be rewritten, as the mentioned variables are not predictors, but rather dependent variables in your models.
*Thank you, we have changed this to refer to them as ‘variables’.*

L261-262. “as some observations were recorded during the same bout of grooming or play” -> I find a bit problematic that you used same “trigger” events for multiple focal individuals, and then excluded event ID from the model, because effectively you treated these datapoints are independent which they were not. It would be important to mention how many focal observations come from the same grooming/play event in your dataset.

*Thank you for flagging this point. Although we did originally include event ID, we found that because the majority of datapoints in our dataset were from independent bouts of grooming/play, including this term caused the model to destabilize, and therefore we felt it was acceptable to remove this term in the final model.
 We entirely agree however this is an important aspect to mention, and have added the figures in to the manuscript here:* ‘*In Models 1.1 and 2.1, event ID de-stabilised the model and caused convergence issues, due to the majority of datapoints not having a repeated event ID (110 of 159 events in Model 1.1, and 82 of 136 events in Model 2.1)’*

L301-302, L329. Consider rephrasing these questions into short statements or provide a short subtitle instead.
*Thank you- we have rephrased the Question 1 subtitle to ‘Is there a behavioural contagion effect?’, but feel that any shorter statement would lack clarity.*

L330-331. I would suggest placing the models in the main manuscript, and not supplement (i.e., at least the models showing the significant social closeness and age effects).
*Thank you for this suggestion. We are concerned that including the models in the main manuscript will compromise the text’s readability and accessibility. We feel that by including the main statistics from the model (sample size, significance testing, effect size of significant results), we include adequate detail, and so would prefer to keep the full models in the supplementary materials.*

L333 and elsewhere. A range of how many observations were done per individual is mentioned here, but in the text it is unclear what the “range” refers to; please add this information.
*Thank you- we have reworded this to include e.g. (‘1-12 observations per individual’).*

When reporting findings in the models, remind the reader what were the main and control predictors in the models, and what were the dependent variables.
*Thank you for this suggestion. We have added in a sentence to each relevant section, for example:
‘We modelled the effect of four predictor variables (age, sex, rank, social closeness) and four control variables (time of day, grooming tendency, number of other individuals present, number of grooming bouts observed) on the dependent variable of presence of grooming initiation’. (lines 354-356)*

Figure 5. Here, the differences in play are reported for age categories, but it is not clear whether the age was treated as continuous variable or as a category in the other analyses. Please add this information.
*Thank you for pointing out this confusion. We have added a sentence to the figure legend, explaining that categorization is just for visual clarity:
‘This categorisation is for visualisation purposes, but data were analysed with age as a continuous variable.’*

L349-351; 372-373. As the full models did not have a significantly better fit than the null model, the results need to be treated with caution.
*Thank you for highlighting this. For this reason, we have just commented on the lack of significant results in these models, and avoided any further interpretation of individual effects. We have added a sentence to each model, emphasizing that results should be treated with caution (lines 374, 405).*

L405-408. Would suggest omitting the part with “jealousy” as it is anthropomorphic.
*Thank you. We are keen to include this point as a possible explanation for the effect, and believe it is not overly anthropomorphic as the presence of jealous reactions in chimpanzees has been previously studied (Webb et al., 2020). However, as there may be controversy over the use of the term jealousy, we have softened the wording of these sentences (‘a reaction akin to jealousy’ ‘a reaction resembling jealousy’).*

Final paragraph could be shortened a bit (L466-476).

*Thank you for this point. We have removed the second sentence recapping some of the literature mentioned in the introduction.*

Please indicate somewhere in the manuscript that the data are made freely available, and the address where the reader can find it (this is also a requirement from the journal).

*Thank you for this point. The data are available on figshare, which we have linked and specified in the data availability statement:
 (“All relevant data files are available from the figshare database:* [*https://doi.org/10.6084/m9.figshare.25314394.v1*](https://doi.org/10.6084/m9.figshare.25314394.v1) *(DOI: 10.6084/m9.figshare.25314394)”).*

References should be sorted so that the Latin names of species are written in italic typeface, and that all journal names are capitalized. Furthermore, L557, Ref 33: Delete “Official Journal of the American Society of Primatologists”, L622-623, Ref 61: Please double-check the details as they do not seem accurately reported.


Copy-Edits.
L21, L378. Add that these were captive chimpanzees.
L22. “social closeness bias” -> delete “bias” as it is unclear
L23. “negative” -> would suggest to put it in quotation mark, or write something like “negatively valenced”
L25, L30. “foundational” -> do you mean “fundamental”?
L53. Please add “some primates” as play face is not exhibited by all primates.
L54. “solo play” -> do you mean “solitary play”?
L55. “collaboration” -> do you mean “interaction”?
L55. “social play (hereafter: play)” -> If only from hereafter it should be written as “play”, then include “social” beforehand in lines L49-55.
L95. “this suggests that this” -> “this suggests that it”
L100. “we sought to establish whether in chimpanzees there is a contagion effect for” -> consider replacing with “we sought to establish whether chimpanzees show contagion for”
L104, L135 . “hypothesis” -> “prediction”
L170. “trigger” -> perhaps exchange to “event”
L171. “in front of their face” -> “in front of the observer’s face”
L174. “all focals” -> sounds a bit colloquial, would suggest rephrasing to “all behaviour”
L183. “around them” -> “radius”
L185. “videos” -> “focals”
L187. “there were the lowest amount” -> “there was the lowest amount”
L190. “social scan observations” -> “scan samplings”
L194. “were visually recorded” -> “were recored”
L240, 241. Consider deleting “and then also” and “if not” for clarity.
L287, 289. Delete “then”
L384, L459-461. Please add quotation marks for “catch”.
L401. Delete “only”
Supplementary Appendix 1 is labelled as Supplementary Appendix 2, and Supplementary Appendix 2 is labelled as Supplementary Appendix 3; please change accordingly.

*Thank you so much for such thorough copy edits- we have made changes to all of these as suggested.*

REVIEWER 2

Abstract/Introduction

I’m a bit concerned about the emphasis on this being the “first” evidence of grooming and play contagion in chimpanzees, as the title reads. Other journals recommend avoiding stating results as “firsts,” and I tend to agree. Whether this is (or anything) is a first or not is often argumentative and semantic. Do contagious play faces count as play contagion? Some might argue yes. I also do not understand how auditorily induced grooming (lines 92-3, reference #33) does not count as grooming contagion in chimpnazees. Ultimately, I do not believe results need to be “firsts” to be interesting. This study is plenty compelling whether or not it is a first, so I encourage the authors to consider reframing it without the “first” element.

*Thank you very much for raising this point. We believe this is the first evidence of behavioural contagion of grooming and play behaviour in chimpanzees, with behavioural contagion being defined as when an individual observes a species typical behaviour and then performs a similar behaviour. However, we entirely agree that results do not need to be firsts to be interesting, and so are happy to change the title, and reword the part of the introduction and discussion that frames the study as a first. We have taken out references to ‘first evidence’ in line 20, line 417, and line 507.*

Methods

Line 243: With the PO-MC method, the definitions are whether the focal “initiated” the behavior in the PO or the MC. A literal reading of “initiated” has me worried about the strictness of the data. Initiated means began, so grooming/play in the MC would only be counted by the focal if the focal was not grooming/playing at the beginning of the MC window, but then started during it. Grooming/playing by the focal that commenced just before the MC that carried into the MC would not be counted. The main purpose of the PC-MC method as developed by de Waal and Yoshihara was to control for daily patterns of activity. I.e., did affiliation go up because of the conflict, or because that was the typical time of day when the animals affiliated? For example, if an MC went 10:00-10:05 and the focal started grooming at 9:58 and carried this grooming into the MC, that would not be counted in the data. This would run counter to the whole idea of controlling for time of day. It would also run counter to controlling for time of day if the authors avoided this time period for its MC because the focal was already engaged in grooming. The MC is supposed to be the exact same time of day as the PC, on the nearest subsequent day, regardless of the behaviors the individuals may already be engaged in. If the focal is already grooming/playing, that is precisely what the authors want to know, in addition to whether the behavior starts during the MC. That is the only way to control for activity changes over the course of the day. My concern comes with two elements the authors need to respond to: The first is their methods, and whether they did the MC sessions appropriately. The second is that more detail is needed in the manuscript regarding the selection of MC sessions. I am making some inferences here, so the authors need to expand on their description of their methods.

*Thank you very much for this comment. We felt that it was appropriate for this study to adapt the original PC-MC matching method for several reasons.
Grooming and play occur frequently throughout the day, as opposed to conflict which is a rarer and more spontaneous event, and so we believe exact timing to not be as relevant for the study of these behaviours. Our more flexible method allowed us to add a condition to the protocol of only recording control follows when the focal individual was within 5m of two other individuals, as was necessarily the case in the post-observation follows. Additionally, we could then pair the post-observation and control follows according to approximate number of individuals present. We feel this consideration of the number of surrounding individuals is an important condition to include, when studying the initiation of social interactions.
We did not start control focals if an individual was already engaged in grooming or play, because this was also the case for the post-observation focals (i.e., if an individual was already participating in play and then observed another individual playing, we did not take a post-observation follow, as it could not be determined if their behaviour was influenced by the play they observed, or the play they were already engaging in).
The original PC-MC method is also difficult to practically implement in a sanctuary environment, as the chimpanzees are only sometimes visible at the edge of the large enclosure, and often not at the same time on consecutive days.
We felt that taking control focals opportunistically, and subsequently matching them to post-observation focals, was the best compromise to ensure the goals of the study were met, and believe this to be an unbiased and suitable method for studying the initiation of social behaviours.
We realise that we should have clarified this rationale in the manuscript, and have added explanation in lines 185-187.*

Line 245: Is it appropriate to not include grooming/playing initiated by another individual during the POs and MCs? It takes two to tango, as the saying goes. Receiving grooming/playing may reflect as much of a mood or appetite as initiating, and its that state of initiating or receiving that might be induced by watching others. An individual who does not want to be groomed or to play will simply move away. Perhaps this should be included in the analysis. ‘Grooming/playing received’ could be separated from ‘grooming/playing initiated’, and then both could be combined into ‘grooming/playing total’.

*Thank you very much for this suggestion. We agree that it would be interesting to study the effect on accepting grooming/playing, but we feel that it is outside the scope of this study. Here, we are particularly interested in studying the specific behavioural act of initiation. We expect that behavioural contagion, and what influences this effect, may be expressed differently for proactive initiation vs engaging with others’ initiations, and we think a comparison of these measures would be a very interesting line of future study. We have added in this idea as a suggested future directions (Lines 470-471).*

Lines 305-11 and 318-323: I’m concerned about how this analysis is done. The Wilcoxon compares attracted to dispersed, and it discards the data from the neutral pairs entirely. It is as if the neutral data do not exist. I do not know that that is appropriate. Especially as the last sentence in each paragraph reads “This indicates that individuals were significantly more likely to initiate grooming/play bouts in PO focals than in MC focals.” But, that is not what was tested. More likely to initiate in the PO than MC would include the initiations that also happened in both conditions (i.e., the neutrals). Without designating pairs as attracted, dispersed, or neutral, it would be total initiations in the PO compared to total initiations in the MC. That analysis would match the sentences in the manuscript. It seems to me that the analyses need to change to conform to the comparison the authors want to make, or else the sentences need to change to match the comparisons run.
I think what bothers me about this analysis (even if it is based on prior work) is that the authors want to know if the subjects groom/play more after viewing grooming/playing than their baseline rate of grooming/playing. The baseline rate of both is primarily housed in the neutral pairs. Throwing them out artificially lowers the amount of behavior in the control or baseline condition. The effect may still be significant, but we really should be looking at how much more grooming/playing there is relative to the baseline rate. Throwing out the neutral pairs suggests that the baseline rate of either is near 0, since there is only 1 dispersed pair in each. That magnifies the effect in the statistics (a larger Z score). Instead of what may be a small or moderate increase in grooming/playing, the statistic implies a very large increase. Unless the authors can argue otherwise, some analysis that includes the neutral pairs as part of the baseline rate is needed.

*Thank you very much for highlighting these issues.
 We have reworded this section so that the summary of results found more accurately reflects the specifics of what was tested (e.g. ‘This indicates it was more common for individuals to initiate grooming in the PO and not in the MC than in the MC and not in the PO, providing evidence for a grooming contagion effect’).
We also agree with your point that, as there is a large number of neutral pairs, excluding this data magnifies the statistical significance of the effect. We have instead carried out a Friedman test, with post-hoc pairwise comparisons using Wilcoxon tests with Bonferroni corrections, to compare attracted vs dispersed vs neutral pairs, so as to include all the data(lines 257-258;317-325; 335-343).*

Fig 3a: I do not understand how to interpret this plot. I do not understand what the 8 partitions refers to or why they were done, and I do not understand how to visualize a difference between contagion and no contagion. There are more black dots in and around the bulge in no contagion, which implies more, but more what? Fig 3b: I do not see a legend labeling the white and grey parts of the bars. I also don’t understand what the numbers 1-8 mean in the bars. Are those the 8 segments of data? Why was the data segmented? What does the segmentation represent? I do not see anything in the Methods related to data segmenting.
*Thank you very much for flagging that this. The segmentation was just an attempt to better visualize the data, but we realise this was not useful. We have changed figure 3 to a more simple violin plot, with only the means represented.*

Fig 5: I do not understand why figure 5 does not match figure 3. Shouldn’t they be the same? The analysis of the grooming and contagion data was the same, so the visualization should be as well. Also, why were the grooming data segmented and the play data were not?
*Thank you for this question. We have chosen different plot types for representing the effects of social closeness on grooming contagion (figure 3) and age on play contagion (figure 5) as we believe that for the different effects illustrated, these different plots are the best way to maximise ease of visual interpretation.*

Discussion
Stylistic note: Sometimes when talking about one individual ‘catching’ a behavior of another, scare quotes are used and sometimes they are not. Particularly see the discussion. The authors should be consistent throughout. Either always quotes or never quotes. I also encourage the authors to find a word that does not require scare quotes to make their ideas more precise and less distracting to read.
*Thank you for pointing out this inconsistency. We have removed the scare quotes from the instances we used them.*

Line 445: Is there a way to compare the latencies to groom and play statistically? If they are not statistically different, the authors should not be interpreting the medians as different.
*Thank you for pointing this out. We used a Welch’s t-test to compare the latencies, and have reported this as an exploratory analysis in lines 408-411.
(‘Having observed that play data latencies appeared much shorter than the grooming data latencies, we carried out an exploratory Welch’s T-test, to compare latencies until the initiation of behaviour post-observation. We found that latencies for the play data were significantly shorter than the latencies for the grooming data (t = 2.758, df = 99.9, p < .001).’)*

Line 467-9: The conclusion here seems a bit strong. The authors previously speculated about attention and executive control, but now they are stating it as supported by the evidence. Since they did not directly measure attention or executive control, this sentence is overstating the results. They need to soften the language here.

*Thank you for this point. We have softened the wording as suggested; the sentence now reads ‘the patterns of social contagion presented here are in line with the idea that social contagion may be modulated by both bottom-up attention processes and top-down executive control’.*
